# Efficacy of Dual Glucagon and Glucagon-like Peptide-1 Receptor Agonists Across the Cardiometabolic Continuum: A Review of Current Clinical Evidence

**DOI:** 10.31083/RCM39691

**Published:** 2025-07-28

**Authors:** Panagiotis Stachteas, Athina Nasoufidou, Paschalis Karakasis, Markella Koiliari, Efstratios Karagiannidis, Theocharis Koufakis, Nikolaos Fragakis, Dimitrios Patoulias

**Affiliations:** ^1^Second Department of Cardiology, Aristotle University of Thessaloniki, Hippokration General Hospital of Thessaloniki, 54642 Thessaloniki, Greece; ^2^Second Propedeutic Department of Internal Medicine, Aristotle University Thessaloniki, Hippokration General Hospital of Thessaloniki, 54642 Thessaloniki, Greece

**Keywords:** dual agonists, GLP-1 receptor agonists, glucagon receptor, cardiometabolic diseases, obesity, type 2 diabetes, metabolic dysfunction-associated steatotic liver disease (MASLD)

## Abstract

The global surge in cardiometabolic diseases, including type 2 diabetes, obesity, and cardiovascular diseases, has reached pandemic levels, demanding bold and innovative solutions. Dual glucagon (Gcg) and glucagon-like peptide-1 (GLP-1) receptor agonists represent a groundbreaking advancement in the treatment of this complex and interconnected spectrum of disorders. By harnessing the synergistic power of GLP-1 and Gcg receptor activation, these agents go beyond glucose lowering and weight loss, unlocking new frontiers in energy expenditure, fat oxidation, and liver fat reduction—key targets in conditions such as metabolic dysfunction-associated steatotic liver disease (MASLD). Emerging clinical evidence on agents such as survodutide and cotadutide has revealed striking improvements in glycated hemoglobin (HbA1c) levels and body weight, consistently outperforming traditional GLP-1 receptor agonists. More importantly, early evidence suggests meaningful benefits in cardiovascular and renal outcomes, positioning these therapies as comprehensive, disease-modifying tools for patients with multiple high-risk comorbidities. This review highlights the transformative potential of dual GLP-1/Gcg receptor agonists, providing a thorough examination of their mechanisms of action, clinical efficacy, and safety profiles across the cardio–metabolic continuum. As the limitations of existing therapies become increasingly evident, these next-generation agents are poised to redefine the standard of care across the cardiometabolic continuum, ushering in a new era of precision medicine for metabolic disease.

## 1. Introduction

The global burden of cardiometabolic diseases, including type 2 diabetes (T2D), obesity, and cardiovascular diseases (CVDs), continues to rise exponentially, representing a major public health challenge and a leading cause of morbidity and mortality worldwide, according to the World Health Organization (WHO) [1]. As of 2021, over 537 million adults worldwide (over 10.5% of the adult population) were estimated to be living with T2D, a number projected to increase significantly over the coming decades, reaching 783 million by 2045 [2]. Nowadays, T2D ranks among the top causes of premature death and was responsible for more than 6.5 million deaths in 2021 [3]. Similarly, according to the World Obesity Atlas 2024, launched by the World Obesity Federation, projections for 2035 indicate that over 1.77 billion individuals will be overweight, while 1.53 billion will be obese. This is expected to account for 54% of the global adult population, further fueling the progression of metabolic disorders and associated complications [4].

CVDs, partly driven by metabolic dysfunction and dysregulation, remain the leading cause of death globally and substantially contribute to loss of health, poor quality of life, and excess health system financial costs [5, 6]. Notably, more than 10% of total health expenditure is spent exclusively on cardiometabolic disease management globally [7]. Recently, the COVID-19 pandemic, which significantly disrupted chronic care delivery worldwide and compromised regular medical visits [8], with only a partial recovery mediated by the increased telemedicine use [9], underlined the unmet need of reducing early morbidity due to non-communicable diseases, including cardiometabolic diseases, a goal that was already prioritized in the 2030 WHO Agenda for sustainable development (Target 3.4) [10]. These conditions share common pathophysiological pathways, characterized by insulin resistance, chronic low-grade systemic inflammation, oxidative stress, abnormal lipid accumulation, and endothelial dysfunction, which collectively contribute to their progression [11, 12]. Effective therapeutic strategies that address multiple facets of this continuum are urgently needed.

Incretin-based agents are increasingly being explored across a broader spectrum of cardiometabolic disorders, beyond their well-established roles in T2D and obesity. Emerging evidence suggests that glucagon-like peptide-1 (GLP-1) receptor agonists (GLP-1 RAs) may offer benefits in obstructive sleep apnea by promoting weight loss and potentially reducing upper airway collapsibility [13]. Additionally, type 1 diabetes mellitus (T1DM)—historically managed without incretin-based therapies—is gaining attention as a candidate for adjunctive GLP-1 RA use, with recent studies showing improvements in glycemic variability and insulin requirements [14, 15]. Furthermore, there is preliminary interest in applying these therapies to maturity-onset diabetes of the young (MODY) subtypes, particularly those with features of insulin resistance or hepatic steatosis [16]. These findings underscore the evolving role of incretin-based therapies in addressing the complex interplay of glucose metabolism, adiposity, and inflammation across diverse cardiometabolic phenotypes.

Building on the expanding therapeutic landscape of incretin-based agents, there is growing interest in combination approaches that can address multiple interconnected metabolic abnormalities. Among these, dual GLP-1/glucagon (Gcg) receptor agonists represent a next-generation class designed to leverage the complementary actions of both hormones. Dual Gcg and GLP-1 RAs offer a novel therapeutic approach to tackle the multifaceted challenges of the cardiometabolic continuum. While GLP-1 RAs effectively lower glucose levels and promote weight loss, their effects on energy expenditure and hepatic fat reduction are modest [17]. Thus, by leveraging the additional impact of Gcg receptor activation, dual agonists have the potential to enhance energy expenditure, promote greater fat oxidation, and reduce liver fat, addressing critical unmet needs in conditions such as non-alcoholic fatty liver disease (NAFLD), currently re-named as metabolic dysfunction-associated steatotic liver disease (MASLD) [17]. Clinical studies of dual agonists, such as survodutide and cotadutide, have shown promising results, including superior reductions in glycated hemoglobin A1c (HbA1c) and body weight, compared to GLP-1 RAs. Moreover, these agents hold promise for improving cardiovascular and renal outcomes, providing a comprehensive therapeutic solution for patients with multiple comorbidities [18, 19]. This synergistic mechanism offers a compelling rationale for targeting the full spectrum of cardiometabolic diseases.

This review aims to synthesize the available clinical evidence on the efficacy and safety of dual Gcg and GLP-1 RAs across the cardiometabolic continuum. Specifically, this review explores the impact of dual Gcg and GLP-1 RAs on glycemic control, weight reduction, surrogate cardiovascular outcomes, and comorbid conditions, including MASLD and chronic kidney disease (CKD). Additionally, this review highlights the mechanisms of action, safety profiles, and potential advantages of dual agonists compared to existing therapeutic options. Finally, by addressing the therapeutic gaps in current management strategies, this review seeks to provide insights into the clinical utility and future directions of these promising agents in the management of cardiometabolic diseases.

## 2. Mechanistic Pathways of Glucagon and GLP-1 in the Cardiometabolic Continuum

GLP-1 and Gcg are key incretin and counter-regulatory hormones, respectively, which exert distinct but complementary effects across the cardiometabolic spectrum [20]. The mechanisms of action of these dual agonists involve both metabolic and extrametabolic pathways, making them attractive targets for therapeutic strategies aimed at addressing obesity, T2D, and CVD [20, 21].

GLP-1, an incretin hormone secreted by intestinal L-cells in response to nutrient intake, plays a central role in glucose homeostasis, primarily by enhancing glucose-dependent insulin secretion from pancreatic β-cells, thereby reducing postprandial glycemic excursions [22, 23]. Beyond its insulinotropic effect, GLP-1 also plays a significant role in the gut–brain regulatory axis, with pleiotropic effects on appetite and energy metabolism, extending beyond its peripheral glucoregulatory actions by acting directly on specific areas of the brain [21]. More specifically, GLP-1 delays gastric emptying and exerts anorexigenic effects through hypothalamic pathways, leading to reduced appetite and subsequent weight loss [24]. In addition, GLP-1 receptors are expressed in the cardiovascular system, and the activation of these receptors has been associated with a reduction in blood pressure, improved endothelial function, and cardioprotective effects, including reduced inflammation and oxidative stress [22, 23]. These pleiotropic actions have positioned GLP-1 RAs as cornerstone treatment agents, not only for glycemic control but also for cardiovascular risk reduction in high- and very-high-risk individuals living with T2D and obesity [25, 26, 27].

Gcg, secreted by pancreatic alpha cells, is traditionally viewed as a counter-regulatory hormone that opposes insulin; Gcg also contributes to metabolic regulation. The primary role of Gcg in hepatic glucose output is well established, playing a central role in maintaining blood glucose levels via glycogenolysis and gluconeogenesis in the liver [28]. However, recent evidence highlights the role of Gcg in increasing energy expenditure, mediated in part by the activation of brown adipose tissue and thermogenesis [29]. Through central and peripheral mechanisms, Gcg enhances lipolysis by promoting lipid oxidation, making it a potential target for weight management [28]. Furthermore, the effects of Gcg on hepatic lipid metabolism—including reductions in steatosis—have gained attention in the context of MASLD [30]. Over the past decade, several drugs targeting the preproglucagon gene signaling systems, particularly GLP-1, have been developed for treating T2D and obesity, with increasing interest in Gcg agonists as part of multi-receptor therapies for cardiometabolic diseases [20]. However, the therapeutic potential of targeting the Gcg receptor has been underexplored, largely due to concerns regarding hyperglycemia [31]. However, by combining the complementary mechanisms of GLP-1 and Gcg, these dual agonists aim to address the limitations of single-receptor therapies.

**Fig. 1.**
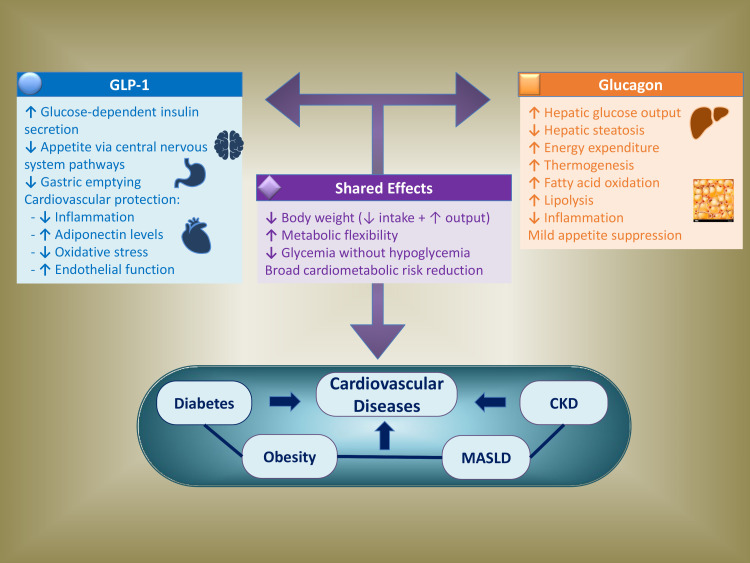
**Complementary mechanisms of GLP-1 and glucagon across the cardiometabolic continuum**. The distinct and overlapping pathophysiological effects of GLP-1 and glucagon support the rationale for dual receptor agonists in the management of cardiometabolic diseases. GLP-1 exerts insulinotropic, anorexigenic, and cardioprotective effects, while glucagon affects white and brown adipose tissue via catabolic and thermogenic pathways, enhancing energy expenditure, lipid oxidation, and hepatic metabolic flexibility. Their synergistic actions contribute to weight loss, improved glycemic control, and reduced risk of hypoglycemia, as well as broad cardiometabolic benefits. CKD, chronic kidney disease; GLP-1, glucagon-like peptide-1; MASLD, metabolic dysfunction-associated steatotic liver disease.

The complementary physiological roles of GLP-1 and Gcg—GLP-1 modulating glycemia and cardiovascular risk, and Gcg enhancing energy expenditure and hepatic metabolic flexibility—form the biological rationale for the development of dual receptor agonists. These agents aim to leverage the benefits of both hormones, potentially overcoming the limitations of monotherapy and offering a more holistic approach to treating cardiometabolic diseases (Fig. 1).

Dual activation of the GLP-1 and Gcg receptors may confer cardiovascular benefits through multiple complementary molecular pathways. GLP-1 receptor signaling enhances endothelial nitric oxide (NO) production by upregulating endothelial nitric oxide synthase (eNOS), leading to improved vasodilation and vascular tone [32, 33]. Additionally, GLP-1 exerts anti-inflammatory effects by inhibiting nuclear factor-kappa B (NF-κB) signaling and reducing the expression of proinflammatory cytokines, such as TNF-α and IL-6, within vascular tissues [34, 35]. Gcg receptor activation may indirectly augment these effects by promoting hepatic and adipose lipid mobilization, thereby lowering ectopic fat accumulation and systemic inflammation [36, 37]. Moreover, preclinical studies suggest that Gcg agonism may modulate macrophage polarization toward an anti-inflammatory M2 phenotype and reduce oxidative stress in cardiac and vascular tissues [38]. Together, these mechanisms may attenuate endothelial dysfunction, atherosclerotic progression, and cardiac remodeling, offering a plausible explanation for the early cardiovascular signals observed with dual agonists, despite limited formal outcome trial data to date.

The differential efficacy of dual GLP-1/Gcg receptor agonists across cardiometabolic diseases can be attributed to variations in disease-specific pathophysiology and tissue receptor expression. For instance, in obesity and MASLD/metabolic dysfunction-associated steatohepatitis (MASH), where energy imbalance and hepatic steatosis are dominant features, the thermogenic and lipolytic effects mediated by Gcg receptor activation provide a complementary mechanism to GLP-1-induced appetite suppression. In contrast, in type 2 diabetes, the insulinotropic and glucagonostatic effects of GLP-1 dominate the glucose-lowering profile; meanwhile, the addition of Gcg activity enhances weight loss and hepatic lipid turnover—two key drivers of insulin resistance. In MASLD, hepatic Gcg receptor stimulation may improve steatosis and metabolic flexibility, whereas in cardiovascular diseases, the anti-inflammatory and endothelial-stabilizing properties of GLP-1 are more critical. These disease-specific pathophysiological differences inform the therapeutic rationale for dual receptor agonism and may guide patient selection in future precision medicine approaches.

## 3. Clinical Evidence of Efficacy Across the Cardiometabolic Continuum

Dual GLP-1 and Gcg RAs offer a promising approach for managing obesity and promoting weight loss by combining the appetite-suppressing effects of GLP-1 RAs with the metabolism-enhancing properties of Gcg. These agents have been extensively studied in both preclinical and clinical settings, demonstrating not only their efficacy in reducing body weight and improving glycemic control but also showing beneficial effects on cardiovascular health, renal function, and MASLD.

### 3.1 Glycemic Control

Survodutide, a dual agonist of the Gcg and GLP-1 receptors, has demonstrated dose-dependent reductions in both HbA1c and body weight over a 16-week treatment period in patients with T2D, compared to placebo. Additional benefits included significant reductions in waist circumference, particularly in patients receiving regimens of 2.7 mg once weekly or 1.8 mg twice weekly [39]. To further assess its potential in obesity—with or without diabetes—the ongoing SYNCHRONIZE™-1 and SYNCHRONIZE™-2 trials are evaluating the long-term efficacy, safety, and tolerability of survodutide in broader patient populations [40].

Another dual-acting agent, cotadutide (GLP-1/Gcg receptor agonist), has shown consistent improvements in glycemic control and body weight reduction in patients with T2D. In a randomized, double-anonymized, Phase 2a study involving overweight or obese individuals with T2D, cotadutide significantly reduced postprandial glucose levels and body weight over a 49-day treatment period compared to placebo [41]. Fasting plasma glucose concentrations and HbA1c levels were also significantly lowered. Glucose-lowering effects were attributed to enhanced insulin secretion and delayed gastric emptying [41]. ​A meta-analysis incorporating data from nine clinical studies confirmed the efficacy of cotadutide in lowering both HbA1c and fasting glucose levels, reinforcing its potential role in managing metabolic disease [42].

Additionally, oxyntomodulin (OXM)—an endogenous gut-derived peptide that co-activates GLP-1 and Gcg receptors—has demonstrated promising metabolic effects. In a small clinical trial, intravenous OXM significantly boosted insulin secretion and mitigated post-infusion hyperglycemia in overweight and obese participants. Importantly, in individuals with T2D, OXM improved β-cell glucose responsiveness, aligning insulin dynamics more closely with those seen in non-diabetic individuals [43].

### 3.2 Weight Reduction and Obesity Management

In the pivotal 46-week trial of survodutide, treatment led to dose-dependent reductions in body weight, with mean weight loss ranging from –6.2% at the 0.6 mg dose to –14.9% at the 4.8 mg dose, compared to –2.8% observed in the placebo group [44]. Moreover, more than 50% of participants treated with 4.8 mg of survodutide achieved a weight loss of 15% or greater [44]. Notably, the benefits were particularly substantial in participants with a baseline body mass index (BMI) ≥27 kg/m^2^, and treatment was well-tolerated across all dose ranges, with a favorable safety profile [45]. A recent meta-analysis of randomized controlled trials further confirmed that survodutide significantly reduced body weight, BMI, and waist circumference compared to the placebo. These effects were more pronounced with higher doses and longer treatment durations [46].

In a 24-week Phase 2 clinical trial, mazdutide, a dual GLP-1 and Gcg RA developed in China, demonstrated significant and dose-dependent reductions in body weight among overweight or obese individuals. Individuals receiving 3 mg, 4.5 mg, and 6 mg doses of mazdutide experienced mean weight losses of –6.7%, –10.4%, and –11.3%, respectively, compared to a weight gain of +1.0% in the placebo group [47]. These findings underscore the potential of mazdutide as a promising therapeutic option for obesity, with efficacy comparable or even superior to that of currently approved GLP-1 RAs. Treatment was generally well tolerated, with the most common adverse events being gastrointestinal and consistent with the incretin-based mechanism of action employed by the drug [47]. Ongoing studies are expected to define the role of mazdutide in managing obesity and dysmetabolism further.

​Clinical studies have demonstrated that cotadutide is effective in promoting weight loss among individuals with overweight or obesity, including those with T2D. In a 54-week randomized Phase 2b study, participants receiving cotadutide experienced significant reductions in body weight compared to the placebo group [48]. Specifically, those treated with cotadutide achieved a mean weight loss of approximately 5.1%, while the placebo group had a weight loss of about 1.2% [48]. Additionally, a meta-analysis of randomized controlled trials (RCTs) assessing the safety and efficacy of GLP-1 and Gcg RAs, including cotadutide, found that these agents significantly reduced body weight and improved glycemic control in individuals with T2D and obesity. The analysis reported a mean percentage reduction in body weight of approximately 4.16% compared to placebo [49].​ This indicates a notable efficacy of cotadutide in weight management for individuals with T2D and obesity. ​However, the development of cotadutide was recently halted by the sponsor pharmaceutical company due to strategic pipeline considerations and not due to any newly observed safety signals or a change in the risk/benefit profile.

Several studies have highlighted the metabolic benefits of OXM—a naturally occurring dual agonist of GLP-1 and Gcg receptors—in populations with obesity or diabetes. In a previous trial, subcutaneous OXM administration resulted in greater weight loss and reduced energy intake compared to the placebo. Additionally, OXM administration was associated with decreased leptin and increased adiponectin levels, suggesting enhanced fat metabolism and improved adipose tissue function [50]. Further RCTs confirmed the ability of OXM to induce a negative energy balance and promote weight reduction in overweight and obese individuals [50], as well as in healthy volunteers [50], suggesting its effect is not limited by metabolic status.

In a 4-week RCT involving patients with obesity and T2D, infusion of a combination of GLP-1, OXM, and Peptide YY (GOP) resulted in significant improvements in glycemic control and moderate weight loss compared to placebo. GOP therapy also reduced fructosamine levels and improved postprandial glucose tolerance. While the magnitude of weight loss was less than that observed with Roux-en-Y gastric bypass or a very low-calorie diet, GOP infusion achieved superior glucose control with lower glycemic variability, highlighting its potential as a non-surgical metabolic intervention [51].

### 3.3 Cardiovascular Outcomes

Robust cardiovascular outcome trials (CVOTs) have consistently demonstrated that GLP-1 RAs reduce major adverse cardiovascular events (MACEs) in patients with T2D and established atherosclerotic disease. Agents such as liraglutide (LEADER), semaglutide (SUSTAIN-6), and dulaglutide (REWIND) have shown significant reductions in cardiovascular death, non-fatal myocardial infarction, and stroke [52]. In contrast, the cardiovascular effects of isolated Gcg receptor agonists are less well established due to their limited development as standalone therapies. However, preclinical studies suggest that Gcg agonism may increase energy expenditure and reduce hepatic steatosis, which could indirectly benefit cardiovascular health, albeit with a theoretical risk of increased heart rate and blood pressure [28, 29]. These findings provide a mechanistic basis for the combined targeting of GLP-1 and Gcg receptors in dual agonist therapies.

While extensive CVOTs have established that GLP-1 RAs reduce the risk of MACEs in patients with T2D [52], specific data on dual GLP-1/Gcg RAs, such as cotadutide and survodutide, are currently limited. Preliminary studies suggest potential cardiovascular benefits; however, comprehensive CVOTs are needed to confirm their effects on cardiovascular morbidity and mortality.​ More specifically, the cardiovascular safety and efficacy of survodutide will be evaluated in the SYNCHRONIZE-CVOT, a Phase 3, randomized, double-anonymized, parallel-group, event-driven, cardiovascular safety study [53]. Regarding blood pressure, a post hoc analysis revealed that survodutide reduced both systolic and diastolic blood pressure in overweight or obese individuals, independent of their baseline history of hypertension, a result consistent with other studies on the effects of incretin-based mono- and dual-agonists [54].

### 3.4 MASLD and MASH

Recent RCTs have highlighted the therapeutic potential of dual GLP-1 and Gcg RAs in managing MASLD and its progression to steatohepatitis (MASH). In a placebo-controlled study, pemvidutide demonstrated robust efficacy in reducing liver fat content (LFC) in overweight or obese individuals with MASLD [55]. By week 12, all administered doses significantly reduced LFC, with the 1.8 mg group achieving the greatest effect—a 68.5% reduction and normalization of LFC in 55.6% of participants. Improvements in non-invasive biomarkers of liver inflammation, as well as in body weight, were also observed [55].

Similarly, survodutide has shown promise in MASH management. In a 48-week Phase 2 trial involving 293 biopsy-confirmed MASH patients, the treatment achieved MASH resolution without worsening of fibrosis in up to 62% of participants, significantly outperforming the placebo (14%). A ≥30% LFC reduction was observed in 67% of participants, while up to 36% experienced at least one stage of fibrosis improvement [56]. Additional data from a multinational, non-randomized, open-label, Phase 1 clinical study in 82 individuals with Child–Pugh class A, B or C cirrhosis and healthy individuals with or without overweight/obesity matched for age, sex, and weights who received survodutide revealed reductions in liver stiffness, enhanced liver fibrosis (ELF) scores, N-terminal type III collagen propeptide (Pro-C3), and body weight over 28 weeks—particularly in non-cirrhotic and Child–Pugh A patients [57]. The treatment was well tolerated, with no major hepatic or renal adverse events [57]. Based on these findings indicating substantial improvement over available treatments, survodutide has received the United States Food and Drug Administration (FDA) Breakthrough Therapy designation and is advancing to Phase 3 trials for MASH.

Cotadutide has also demonstrated substantial hepatic benefits. In a 41-day Phase 2a trial, cotadutide reduced LFC by 39.1% compared to 19.5% with placebo, likely through hepatic Gcg signaling [58]. A subsequent 54-week Phase 2b trial in 834 individuals with obesity and inadequately controlled T2D under metformin showed improvements in non-invasive MASLD markers with cotadutide administration. However, histological confirmation via biopsy was lacking [48].

A recent meta-analysis identified survodutide and tirzepatide as the most effective agents in achieving MASH resolution, with or without concurrent fibrosis improvement, suggesting a potentially disease-modifying role [59]. While these findings are encouraging, many studies rely on surrogate endpoints or lack histological confirmation. Larger, longer-term trials with liver biopsy data and cardiovascular safety outcomes are essential to fully define the clinical utility of dual receptor agonists in MASLD/MASH and their systemic metabolic impact.

Preclinical data further support the utility of this class. OXM-104, a long-acting oxyntomodulin analog, showed greater reductions in steatosis, fibrosis, and liver injury markers compared to semaglutide, and improved NAFLD activity score (NAS), highlighting its potential in treating liver-related metabolic complications [60]. Additionally, S3-2re, another long-acting OXM analog, demonstrated not only superior metabolic effects compared to liraglutide but also renoprotective properties, reversing diabetic nephropathy in murine models [61].

## 4. Comparison of Dual Agonists With Other Therapeutic Options

Compared to GLP-1 RAs, which have proven efficacy in glycemic control, weight reduction, and cardiovascular risk reduction, dual agonists appear to offer superior effects on weight loss and hepatic endpoints, albeit with limited data on cardiovascular and renal outcomes. The comparative advantage of dual Gcg/GLP-1 RAs lies in their combined mechanism of action. These agents not only suppress appetite and improve glucose regulation but also stimulate energy expenditure and lipolysis through Gcg receptor agonism. Indeed, current clinical data suggest that dual agonists consistently show greater body weight reductions than GLP-1 RAs alone [62, 63]. Survodutide and pemvidutide, for example, have achieved >15% body weight loss in a significant proportion of patients, comparable to the outcomes of tirzepatide and bariatric surgery in some trials [53, 55].

Unlike GLP-1 RAs, dual agonists target both glucose metabolism (via GLP-1) and lipid/energy homeostasis (via Gcg), potentially improving hepatic steatosis, insulin resistance, and metabolic flexibility. Cotadutide and other dual Gcg/GLP-1 receptor agonists have demonstrated reductions in liver fat and fibrosis markers, beneficial in MASLD/MASH, where other agents (except for tirzepatide [64]) show limited efficacy [41, 48].

In contrast, sodium-glucose co-transporter 2 inhibitors (SGLT2is), due to their pleiotropic effects [65, 66, 67], offer robust cardiovascular and renal protection, particularly in heart failure across the spectrum of ejection fraction and CKD; nonetheless, these inhibitors induce modest weight loss and modest glycemic improvements relative to incretin-based therapies [68]. However, despite Gcg receptor activation, most dual agonists maintain a glucose-dependent insulinotropic profile, thereby minimizing the risk of hypoglycemia. Notably, dual Gcg/GLP-1 RAs currently lack data on renal outcomes.

Tirzepatide, a dual glucose-dependent insulinotropic polypeptide (GIP)/GLP-1 agonist, has set a new standard in metabolic control, demonstrating superior weight loss and HbA1c reduction compared to GLP-1 RAs in the SURPASS and SURMOUNT programs [69]. While dual GLP-1/Gcg agonists have achieved similar or even greater weight loss in early-phase studies [63], tirzepatide has the advantage of extensive clinical trial data, including CVOTs, which are currently underway (e.g., SURPASS-CVOT) [70, 71, 72].

Unlike GLP-1 RAs (e.g., semaglutide, liraglutide), SGLT2is (e.g., empagliflozin), and tirzepatide, which have robust CVOT data, dual agonists lack definitive outcome data. Preliminary data indicate favorable effects on blood pressure, lipids, and inflammation, although robust CVOTs are pending. On the contrary, Gcg activity can lead to increased heart rate, raising theoretical concerns about long-term cardiovascular safety, particularly in patients with underlying cardiac conditions [73].

However, dual GLP-1/Gcg RAs also come with potential drawbacks, including gastrointestinal side effects, increased heart rate, and uncertainty regarding long-term safety. Thus, the use of dual GLP-1/Gcg RAs in clinical practice will depend heavily on the outcome of ongoing trials, particularly those assessing cardiovascular and renal outcomes, as well as tolerability. Moreover, balancing the catabolic and hyperglycemic potential of Gcg with the insulinotropic effects of GLP-1 is pharmacologically challenging, and dose titration must be carefully managed to avoid adverse metabolic effects.

Overall, dual GLP-1/Gcg RAs have emerged as a promising therapeutic approach for comprehensive cardiometabolic risk modification, especially in obesity and MASLD/MASH. These dual RAs exhibit enhanced efficacy in reducing weight and liver fat compared to GLP-1 RAs, and may complement the cardioprotective benefits of SGLT2 inhibitors in future combination strategies. However, until long-term cardiovascular and renal data are available, these agents remain in the background, and their clinical positioning relative to GLP-1 RAs, SGLT2is, and tirzepatide should be approached with cautious optimism (Table 1).

**Table 1.** **Comparison of dual agonists with other therapeutic options**.

	GLP-1 RAs	SGLT2is	Tirzepatide	Dual GLP-1/glucagon agonists	Pioglitazone	Retatrutide
HbA1c reduction	High	Moderate	Very high	High	Moderate	Very high
Weight loss	Moderate	Mild	Very high	Very high	Mild weight gain	Very high
Entry 2 CV outcome trials (MACEs)	Completed, benefit shown	Completed, benefit shown	Ongoing	Not yet available	Completed, neutral	Not yet available
HF outcomes	Moderate benefit (esp. HFpEF)	Strong benefit (HFrEF, HFpEF, CKD)	Moderate benefit (esp. HFpEF)	Limited data	Increased risk (esp. in HFrEF)	Limited data
Lipid profile improvements	Mild (↓TG, ↑HDL)	Mild (↓TG, ↓small dense LDL)	Moderate	Moderate to strong (↓TG, ↓hepatic fat)	↑HDL, ↑TG	↓TG, ↓hepatic fat
Liver fat and MASH/MASLD impact	Mild improvement	Inconclusive	Moderate (improved steatosis)	Strong effects on LFC, MASH resolution, fibrosis improvement	Strong histologic improvement	Promising surrogate data
GI side effects	Common (nausea, vomiting)	Rare	Common (nausea, vomiting)	Often more frequent/severe than GLP-1 RAs	Rare	Common
Heart rate increase	Mild	Neutral	Mild	Moderate (via glucagon receptor activation)	Neutral	Moderate
Hypoglycemia risk	Low (monotherapy)	Low	Low	Low	Low	Low
Renal protection	Moderate	Strong	Moderate (data pending)	Limited evidence	None	Not yet available
Tolerability and discontinuation	Moderate	High	Moderate-high	Possibly lower due to GI burden	Moderate (fluid retention)	Moderate (GI burden)

CKD, chronic kidney disease; CV, cardiovascular; GI, gastrointestinal; GLP-1 RAs, glucagon-like peptide-1 (GLP-1) receptor agonists; HbA1c, glycated hemoglobin A1c; HDL, high-density lipoprotein; HF, heart failure; HFpEF, HF with preserved ejection fraction; HFrEF, HF with reduced ejection fraction; LDL, low-density lipoprotein; LFC, liver fat content; MACEs, major adverse cardiovascular events; MASH, metabolic dysfunction-associated steatohepatitis; MASLD, metabolic dysfunction-associated steatotic liver disease; SGLT2is, sodium-glucose co-transporter 2 inhibitors; TG, triglycerides.

## 5. Safety and Tolerability

The advent of dual and triple RAs targeting GLP-1, Gcg, and other gut-derived hormones has marked a significant advancement in the pharmacotherapy of obesity and T2D. These agents have demonstrated superior efficacy in inducing weight loss and improving glycemic control through complementary mechanisms of action. However, this therapeutic enhancement is accompanied by a notable increase in treatment-emergent adverse events, predominantly gastrointestinal (GI) disturbances, such as nausea, vomiting, and diarrhea, as well as injection site reactions. Early clinical development of several dual agonists was discontinued due to an unfavorable tolerability profile, especially the high incidence and severity of GI adverse events. Nevertheless, ongoing development efforts focus on refining pharmacokinetics and receptor selectivity to improve the therapeutic index and mitigate side effects. Some analogs remain in clinical development, aiming to balance maximal metabolic efficacy with acceptable tolerability profiles [74].

Safety data primarily stem from Phase 2 trials. Cotadutide was generally well tolerated, with a safety profile consistent with that of established GLP-1 receptor agonists. Dose-dependent gastrointestinal events, especially nausea and vomiting, were the most commonly reported adverse effects [58]. Survodutide demonstrated a higher frequency of adverse events compared to placebo, with gastrointestinal intolerance, including nausea, diarrhea, and vomiting, being the predominant cause of treatment discontinuation in some cases [44, 56]. In contrast, mazdutide was reportedly well-tolerated across all dosing regimens, although apart from the expected GI symptoms, a higher incidence of upper respiratory tract infections was observed [47].

The risk of hypoglycemia with dual agonists remains relatively low, particularly in the absence of concomitant insulin or insulin secretagogues. This is largely attributed to the glucose-dependent insulinotropic action of the GLP-1 component [75]. Nonetheless, the Gcg receptor agonism theoretically raises concerns regarding potential hyperglycemia, necessitating careful dose optimization and monitoring [31].

Long-term safety data are currently lacking, representing a critical gap in the clinical translation of these agents. While short-term studies have not raised major safety signals, animal studies and secondary analyses of clinical trials have raised concerns regarding a potential association with acute pancreatitis, C-cell hyperplasia, and medullary thyroid carcinoma—risks that are also recognized with conventional GLP-1 RAs [76, 77]. Additionally, the long-term impact of dual agonists on cardiovascular outcomes, renal function, and other organ systems remains under investigation [59, 62, 74].

Regarding long-term safety, further studies are necessary. Moreover, some concerns have been noted in animal studies and secondary outcomes of clinical studies, particularly referring to the potential risk of pancreatitis and thyroid-related comorbidities, which have also been observed with GLP-1 RAs. Additionally, long-term outcomes related to cardiovascular health, renal function, and other organ systems are still being evaluated.

Lastly, concerns have arisen about the loss of lean muscle mass with the widespread use of GLP-1 RAs [78, 79, 80]. This raises questions about the overall safety of GLP-1-based agents, and it remains to be determined in future trials whether the true impact of dual Gcg/GLP-1 RAs on lean mass and related outcomes can be established among eligible participants [78, 79, 80].

In summary, while dual GLP-1/Gcg RAs exhibit a generally acceptable safety profile in early-phase studies, the high prevalence of GI adverse events and unresolved questions regarding long-term safety warrant cautious interpretation. Further large-scale, long-duration trials are essential to fully establish the benefit–risk profile of this promising drug class.

## 6. Future Directions and Unmet Needs

While dual GLP-1/Gcg RAs represent an exciting frontier in cardiometabolic therapeutics, several key challenges and research gaps remain. Combination strategies, such as triple agonists targeting GLP-1, Gcg, and GIP, or adjunctive use with SGLT2is or anti-inflammatory and anti-fibrotic agents, may offer synergistic benefits; however, the safety, tolerability, and real-world applicability of these strategies remain largely unexplored. Furthermore, the process of identifying the patients who are most likely to benefit from dual agonist therapy remains limited. Current studies rely on broad metabolic phenotypes (e.g., BMI, HbA1c), but lack precision medicine tools such as genetic, metabolic, or inflammatory biomarkers that could stratify responders and mitigate risks, especially in high-risk populations with coexisting conditions, such as MASLD or heart failure.

Despite promising short-term data in obesity, T2D, and MASLD, there is a paucity of long-term outcome trials addressing their efficacy on surrogate endpoints, including cardiovascular and renal morbidity and mortality, and all-cause mortality. Ongoing trials, such as SYNCHRONIZE-CVOT, and studies in advanced MASLD/MASH populations, are expected to clarify the benefit–risk profile of these agents. Data derived from these studies will likely inform future guideline recommendations and potentially redefine treatment algorithms across the cardiometabolic spectrum. Nonetheless, questions remain regarding the durability of weight loss, the optimal balance of GLP-1 vs. Gcg activity, and the cardiovascular implications of Gcg-mediated heart rate elevation. Ultimately, this field must shift toward mechanistically informed, patient-tailored strategies to fully realize the potential of dual agonists in the comprehensive management of cardiometabolic diseases.

## 7. Conclusions

Dual GLP-1 and Gcg RAs represent a promising advancement in the management of cardiometabolic diseases, particularly obesity, T2D, and MASLD/MASH. Further, these agents might offer enhanced metabolic benefits compared to single GLP-1 RAs by combining the glycemic, anorectic, and cardioprotective actions of GLP-1 with the thermogenic and lipolytic effects of Gcg. Early clinical data suggest superior efficacy in weight loss and hepatic outcomes, with some potential additive cardiovascular benefits, although this is based on preliminary evidence. However, the long-term safety and efficacy of these dual agents remain uncertain due to the limited availability of relevant trial data. Thus, while these agents hold potential to reshape treatment paradigms, their clinical adoption should remain cautious.
